# The Effects of Breastfeeding on Childhood Behavioral and Emotional Development: A Prospective Cohort Study in China

**DOI:** 10.3390/nu16111743

**Published:** 2024-06-02

**Authors:** Ying Meng, Hongzhao Yu, Mingxuan Zhang, Hongtian Li, Yubo Zhou, Jianmeng Liu

**Affiliations:** 1 Institute of Reproductive and Child Health/National Health Commission Key Laboratory of Reproductive Health, Peking University Health Science Center, 38 Xueyuan Rd., Beijing 100191, China; mengying@pku.edu.cn (Y.M.); bright@bjmu.edu.cn (H.Y.); zmxemma@163.com (M.Z.); liht@bjmu.edu.cn (H.L.); 2 Department of Epidemiology and Biostatistics, School of Public Health, Peking University Health Science Center, 38 Xueyuan Rd., Beijing 100191, China

**Keywords:** breastfeeding, behavioral outcomes, emotional outcomes, children, cohort study

## Abstract

Background: Breastfeeding could improve a child’s health early on, but its long-term effects on childhood behavioral and emotional development remain inconclusive. We aimed to estimate the associations of feeding practice with childhood behavioral and emotional development. Methods: In this population-based birth cohort study, data on feeding patterns for the first 6 mo of life, the duration of breastfeeding, and children’s emotional and behavioral outcomes were prospectively collected from 2489 mother–child dyads. Feeding patterns for the first 6 mo included exclusive breastfeeding (EBF) and non-exclusive breastfeeding (non-EBF, including mixed feeding or formula feeding), and the duration of breastfeeding (EBF or mixed feeding) was categorized into ≤6 mo, 7–12 mo, 13–18 mo, and >18 mo. Externalizing problems and internalizing problems were assessed with the Child Behavior Checklist (CBCL) and operationalized according to recommended clinical cutoffs, corresponding to *T* scores ≥64. Multivariable linear regression and logistic regression were used to evaluate the association of feeding practice with CBCL outcomes. Results: The median (interquartile range) age of children at the outcome measurement was 32.0 (17.0) mo. Compared with non-EBF for the first 6 mo, EBF was associated with a lower *T* score of internalizing problems [adjusted mean difference (aMD): −1.31; 95% confidence interval (95% *CI*): −2.53, −0.10], and it was marginally associated with *T* scores of externalizing problems (aMD: −0.88; 95% *CI*: −1.92, 0.15). When dichotomized, EBF versus non-EBF was associated with a lower risk of externalizing problems (a*OR*: 0.54, 95% *CI*: 0.34, 0.87), and it was marginally associated with internalizing problems (a*OR*: 0.75, 95% *CI*: 0.54, 1.06). Regarding the duration of breastfeeding, breastfeeding for 13–18 mo versus ≤6 mo was associated with lower *T* scores of internalizing problems (aMD: −2.50; 95% *CI*: −4.43, −0.56) and externalizing problems (aMD: −2.75; 95% *CI*: −4.40, −1.10), and breastfeeding for >18 mo versus ≤6 mo was associated with lower *T* scores of externalizing problems (aMD: −1.88; 95% *CI*: −3.68, −0.08). When dichotomized, breastfeeding for periods of 7–12 mo, 13–18 mo, and >18 mo was associated with lower risks of externalizing problems [a*OR* (95% *CI*): 0.96 (0.92, 0.99), 0.94 (0.91, 0.98), 0.96 (0.92, 0.99), respectively]. Conclusions: Exclusive breastfeeding for the first 6 mo and a longer duration of breastfeeding, exclusively or partially, are beneficial for childhood behavioral and emotional development.

## 1. Introduction

Childhood behavioral and emotional development is a public health concern [1] since its dysregulations could increase the risk of developing mental health problems during adulthood [2,3]. Behavioral and emotional dysregulation among school-aged children has an average multinational prevalence of 9% (range, 2−18%; *n*  =  56,666) [4]. Approximately 10% of American children younger than 5 y experienced clinically significant emotional and behavioral problems [5], and the corresponding prevalence among school-aged children was 15.9% in China [6]. Behavioral and emotional development is influenced by various biological, social, and environmental factors [7,8,9]. Identifying modifiable factors in early life provides opportunities for early intervention and the prevention of behavioral and emotional problems.

The feeding practices of infants may be among the potential modifiable factors for behavioral and emotional problems. The World Health Organization (WHO) recommends exclusive breastfeeding (EBF) for the first 6 months (mo) of life, with the introduction of appropriate complementary foods thereafter and continued breastfeeding up to 2 y of age or beyond [10]. Attributed to the abundant vital nutrients in breastmilk [11] and mother–infant interaction during breastfeeding [12], breastfeeding confers a wide range of benefits for infants [13], such as lower risks of asthma, obesity, diabetes, and leukemia in later life [14]. Accumulating evidence suggests that the feeding practices of infants may affect offspring’s cognitive performance. A meta-analysis of 17 studies showed that breastfeeding was related to an improved intelligence quotient (IQ) among children and adolescents [15]. Although the association between breastfeeding and children’s IQs has been well studied, studies on breastfeeding and offspring’s behavioral and emotional development are limited.

There are only two studies that have assessed the associations of EBF with offspring behavioral and emotional development. A cohort study involving American children showed that ≥3 mo of EBF combined with ≥6 mo of breastfeeding was associated with better emotional and conduct performance at 6 y old compared to children who were never breastfed [16]. The other cohort study, involving Chinese children, found a modest but significant lower score of emotional problems at 5 y old in those who underwent EBF versus non-EBF, whereas no results were reported for behavioral problems [17]. Several studies were conducted to explore the effect of breastfeeding duration on offspring behavioral and emotional development but reported inconsistent results. Some found that a longer duration of breastfeeding was associated with better childhood behavioral and emotional development [18,19,20,21,22], whereas others did not find significant associations [16,23]. In addition, these studies focused on breastfeeding within the first 12 mo of life, and little is known about breastfeeding duration up to 1–2 y of age, as recommended by the WHO. Furthermore, all of these studies treated breastfeeding duration as a categorical variable, and few have explored its relationship as a continuous variable with behavioral and emotional development. Most studies were restricted to a Caucasian population, and the data are sparse for Asian populations, especially Chinese populations [24,25].

The objective of this study was to estimate the associations of EBF as well as a duration of breastfeeding prolonged to >18 mo with childhood behavioral and emotional development using data from a large prospective cohort study in China. Simultaneously, we tried to explore whether the associations varied based on children’s sex.

## 2. Materials and Methods

### 2.1. Participants and Study Design

**Figure 1:**
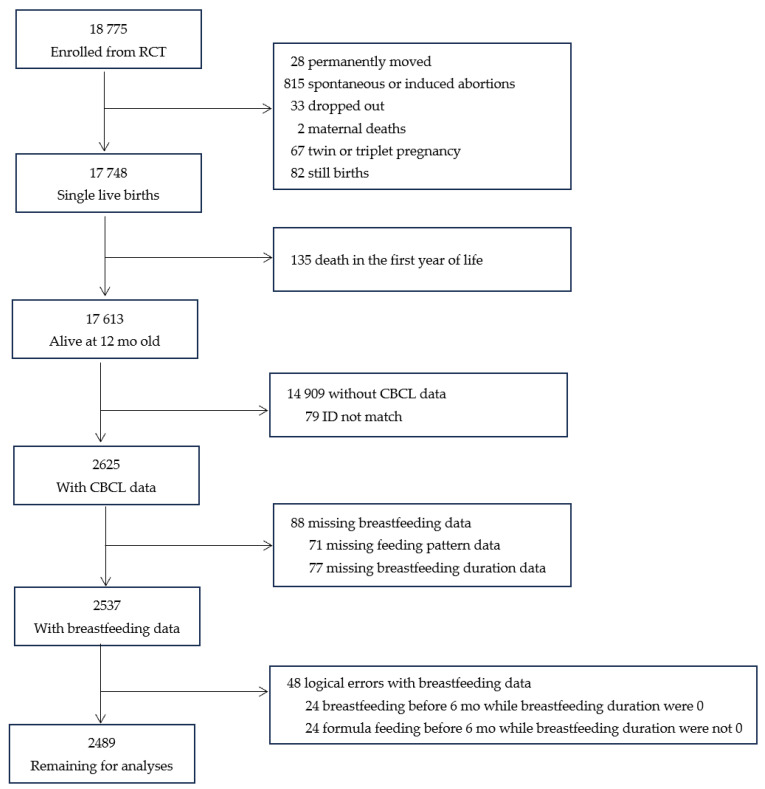
Participant flow chart.

This prospective cohort study was conducted based on a randomized controlled trial (RCT) implemented from May 2006 to April 2009 in Hebei Province, China. The details of the RCT have been provided elsewhere [26]. In brief, 18,775 singleton nulliparous women in early pregnancy were enrolled and randomly assigned to receive folic acid, iron–folic acid, or multiple micronutrients daily until delivery, and their infants were followed up with until 12 mo after birth. Among the 17,613 infants alive at 12 mo old, 2715 children were randomly selected to participate in a follow-up program conducted from May to August in 2011 when they were 18–60 mo old. In this follow-up program, information on duration of breastfeeding and children’s emotional and behavioral development was collected. In this study, we excluded 136 children who had incomplete (*n* = 88) or incorrect (*n* = 48) information on feeding practice, leaving 2489 children in the final analysis (Figure 1). Maternal and children characteristics were comparable between the included (*n* = 2489) and excluded children (*n* = 15,124). (Table A1).

Both the RCT and the follow-up study were approved by the Peking University Health Science Center Institutional Review Board with identifiers of NCT00133744 and NCT01404416 on Clinicaltrials.gov, respectively. All caregivers of the participants provided written informed consent.

### 2.2. Exposure, Outcomes, and Covariates

The exposure variables for the present study were the feeding pattern for the first 6 mo of life and the duration of breastfeeding until the follow-up in 2011. The feeding patterns for the first 6 mo were extracted from the RCT dataset and were categorized as EBF, formula feeding, or mixed feeding. EBF was defined as breastfeeding only without other liquids or solid food [27]. Due to the limited numbers of formula feeding and mixed feeding, they were combined in the non-EBF group in the analysis below. Duration of breastfeeding (EBF and mixed feeding) was used as a continuous variable and a categorical variable, as detailed in the following statistical analysis.

The outcomes, including a range of behavioral and emotional problems, were assessed using a validated Chinese version of the Child Behavior Checklist for ages in the range of 1.5–5.0 y (CBCL 1.5–5.0) [28]. The CBCL, completed by caregivers under the guidance of trained doctors, consists of 99 closed items describing behaviors exhibited by the child during the preceding 2 mo. Each closed item scored 0 (not true), 1 (somewhat or sometimes true), or 2 points (very true or often true). Subscores were calculated for (1) seven syndromes, including emotionally reactive, anxiety/depression, somatic complaints, withdrawn behavior, sleep problems, attention problems, and aggressive behavior; (2) three composite problem scales: emotional or internalizing problems (emotionally reactive, anxious/depressed, somatic complaints, and withdrawn behavior), behavioral or externalizing problems (attention problems and aggressive behavior), and total problems; (3) five scales were oriented to the following Diagnostic and Statistical Manual of Mental Disorders (5th edition) (DSM-V) categories: affective problems, anxiety problems, pervasive developmental problems, attention deficit/hyperactivity problems, and oppositional defiant problems. A normalized *T* score was assigned for each scale according to a normative sample [28]. The behavioral and emotional problems were operationalized according to recommended clinical cutoffs, corresponding to *T* scores ≥64 for the three composite problems and *T* scores ≥70 for seven syndromes and five DSM-V-oriented problems.

Most covariates were extracted from the RCT database, including maternal age at delivery (y), gestational age (week, wk), education (primary school, middle school, or high school or above), occupation (farmer or others), height, weight in early pregnancy, anemia in mid-pregnancy (hemoglobin concentration <110 g/L [29]), supplementation during pregnancy (folic acid, iron–folic acid, or multiple micronutrients), delivery mode (vaginal delivery or cesarean delivery), children’s sex (boy or girl), and birthweight (g). Maternal body mass index (BMI) was calculated as the maternal weight in kilograms divided by height in meters squared and categorized as <18.5, 18.5–22.9, 23.0–27.4, or ≥27.5 kg/m^2^ according to criteria for Asians [30]. Information on the main caregivers of the child (parents or grandparents), kindergarten attendance (yes or no), and child’s age at follow-up (mo) were collected in the follow-up program.

### 2.3. Statistical Analyses

Continuous variables were presented using the mean (standard deviation, SD) or median (interquartile range, IQR) depending on the distribution’s normality assessed by the Shapiro–Wilk test. Categorical variables were presented using frequency (%). Differences between groups of feeding patterns for the first 6 mo of life (EBF and non-EBF) were analyzed by Welch *t*-test for means since the Bartlett test indicated variance heterogeneity between groups, Mann–Whitney test for medians, and chi-square test or Fisher’s exact test for frequencies.

**Figure A1:**
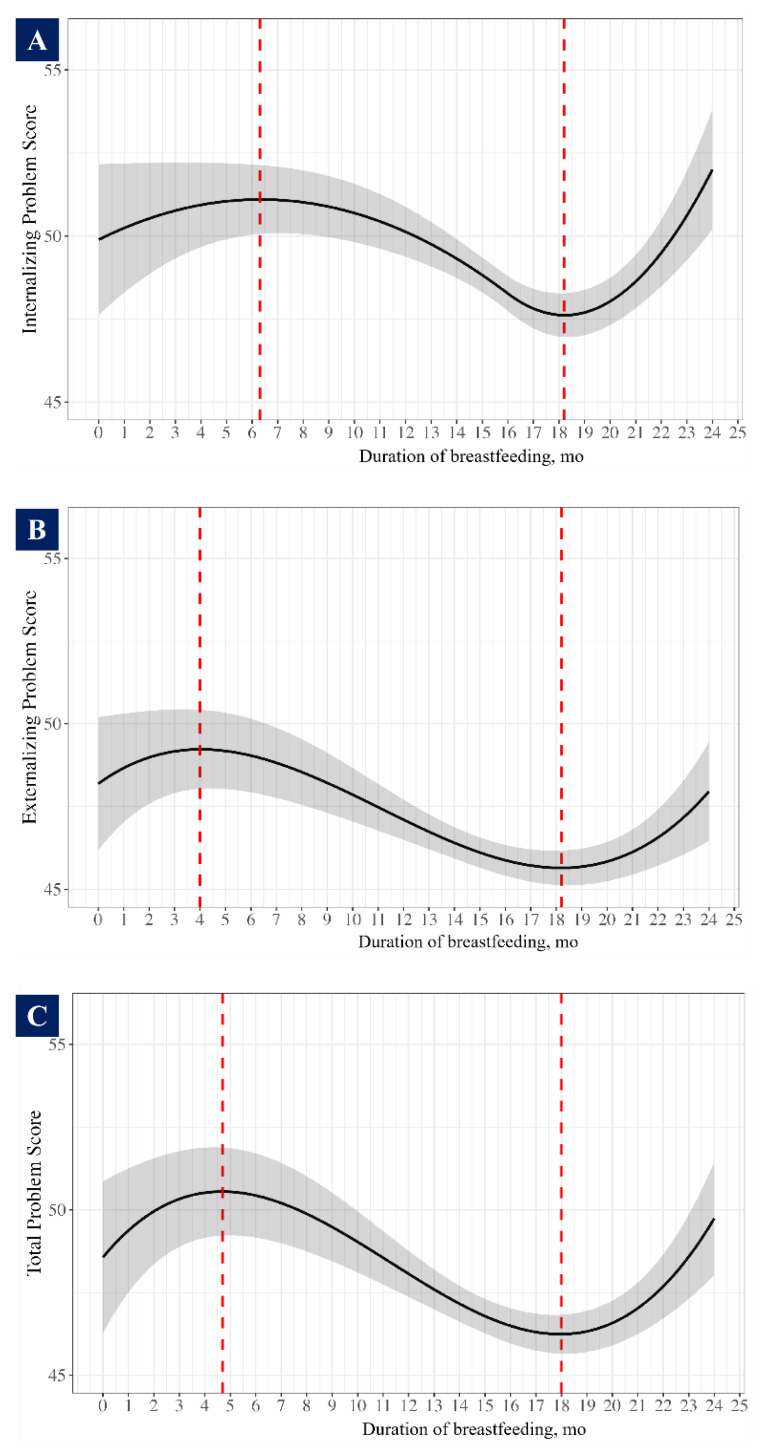
Restricted cubic spline curves depicting a non-linear relationship between the duration of breastfeeding and *T* scores of internalizing problems (**A**), externalizing problems (**B**), and total problems (**C**). Notes: Adjusted for maternal age, gestational week, education, occupation, BMI in early pregnancy, anemia in mid-pregnancy, supplementation during pregnancy, delivery mode, children’s sex, age, birthweight, main caregiver, and kindergarten attendance. The black solid line represents the fitted curve, and the gray area denotes the 95% confidence interval of the fitted values. The red dashed line indicates the location of the inflection point.

Duration of breastfeeding was first used as a continuous variable in multivariable restricted cubic spline (RCS) models to explore its linear or non-linear relationship with *T* scores of behavioral and emotional problems. If a non-linear relationship was observed, a 10-fold cross-validation approach was used to identify the optimal degree of freedom that yielded the highest R-squared value. Then, the inflection points of the constructed curves were determined (Figure A1). On the basis of the inflection points and to make comparisons with previous studies, the duration of breastfeeding was categorized as ≤6 mo, 7–12 mo, 13–18 mo, and >18 mo in the following analysis.

Univariable and multivariable linear regression models were used to calculate the crude mean differences (cMDs) and adjusted mean differences (aMDs) in *T* scores across different groups of feeding patterns for the first 6 mo and on the duration of breastfeeding. Univariable and multivariable logistic regression models were used to estimate the crude odds ratios (c*ORs*) and adjusted odds ratios (a*ORs*) with 95% confidence intervals (95% *CI*) for behavioral and emotional problems. In multivariable analyses, we adjusted for maternal age, gestational age, education, occupation, BMI in early pregnancy, anemia in mid-pregnancy, supplementation during pregnancy, delivery mode, children’s sex, birthweight, main caregiver, kindergarten attendance, and child’s age at outcome measurement.

Because the emotional and behavioral development of children differs based on their sex [31], we performed a subgroup analysis stratified by children’s sex (boys and girls) and evaluated whether the associations varied by children’s sex by adding an interaction term into the multivariable regression models.

All *p*-values were 2-sided, and statistical significance was set at *p* < 0.05. All analyses were conducted using R software (version 4.2.3).

## 3. Results

### 3.1. Participant Characteristics

The median (IQR) maternal age at delivery was 22.7 (3.0) years. A total of 98.8% of the mothers were of Han ethnicity, 99.0% possessed middle school education or above, and 93.5% were farmers. The median (IQR) age of the children at the outcome measurement was 32.0 (17.0) mo. Of the 2489 children, 51.8% were boys, 83.8% underwent EBF for the first 6 mo of life, and their median (IQR) duration of breastfeeding was 16.0 (6.0) mo. The maternal and children characteristics, according to the feeding patterns for the first 6 mo of life, are presented in Table 1. Compared with children who underwent EBF, non-EBF children were more likely to be boys (50.7% vs. 57.4%), to be taken care of by their grandparents (17.8% vs. 34.4%), and to have attended kindergarten (25.3% vs. 30.4%).

### 3.2. Feeding Patterns for the First 6 mo and Behavioral and Emotional Development

According to a univariable analysis, EBF children versus non-EBF children had statistically significant lower *T* scores of internalizing problems (cMD: −1.39; 95% *CI*: −2.59, −0.20), externalizing problems (cMD: −1.33; 95% *CI*: −2.36, −0.30), and total problems (cMD: −1.54; 95% *CI*: −2.71, −0.37) (Table 2). After a multivariable adjustment, the EBF children had significantly lower *T* scores of internalizing problems (aMD: −1.31; 95% *CI*: −2.53, −0.10) and total problems (aMD: −1.26; 95% *CI*: −2.44, −0.08) and marginally higher *T* scores of externalizing problems (aMD: −0.88; 95% *CI*: −1.92, 0.15). EBF was associated with lower *T* scores regarding somatic complaints (aMD: −1.26; 95% *CI*: −1.93, −0.59), aggressive behavior (aMD: −0.57; 95% *CI*: −1.08, −0.06), and DSM-V-oriented anxiety problems (aMD: −0.79; 95% *CI*: −1.47, −0.10). Multivariable logistic regression models showed that EBF was associated with lower risks of externalizing problems (a*OR*: 0.54; 95% *CI*: 0.34, 0.87), and it was marginally associated with lower risks of internalizing problems (a*OR*: 0.75; 95% *CI*: 0.54, 1.06) and total problems (a*OR*: 0.69; 95% *CI*: 0.47, 1.01), as well as with DSM-V-oriented attention deficit/hyperactivity problems (a*OR*: 0.35; 95% *CI*: 0.18, 0.69) (Table 3). No other behavioral or emotional scales were significantly associated with feeding patterns.

### 3.3. Duration of Breastfeeding and Behavioral and Emotional Development

Non-linear relationships were exhibited between breastfeeding duration continuum and *T* scores of internalizing problems, externalizing problems, and total problems. The shapes of their curves were similar, with consistent inflections at around 6 mo and 18 mo. The *T* scores significantly decreased during the 6–18 mo period, but not for ≤6 mo or >18 mo (Table A2).

**Figure 2:**
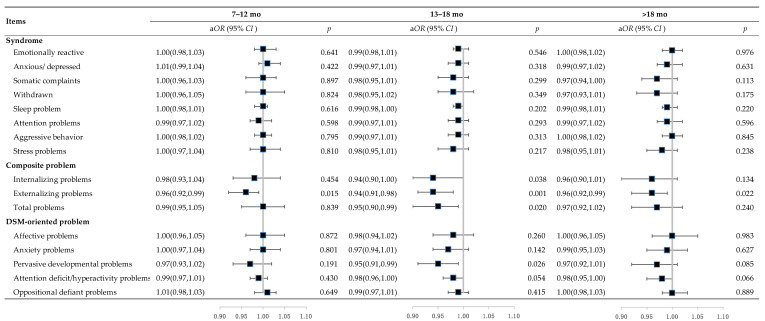
Risks of child behavioral and emotional problems based on duration of breastfeeding, a*OR* (95% *CI*). Notes: Adjusted for maternal age, gestational week, education, occupation, BMI in early pregnancy, anemia in mid-pregnancy, supplementation during pregnancy, delivery mode, children’s sex, age, birthweight, main caregiver, and kindergarten attendance.

Analyses of breastfeeding duration categories showed that, compared with children who were breastfed for ≤6 mo, those who were breastfed for 7–12 mo had no significant differences in behavioral or emotional *T* scores, whereas those who were breastfed for 13–18 mo had significantly lower *T* scores of internalizing problems (aMD: −2.50; 95% *CI*: −4.43, −0.56), externalizing problems (aMD: −2.75; 95% *CI*: −4.40, −1.10), and total problems (aMD: −2.96; 95% *CI*: −4.84, −1.07), and those who were breastfed for >18 mo had lower *T* scores of externalizing problems (aMD: −1.88; 95% *CI*: −3.68, −0.08) (Table 4). In addition, children who were breastfed for 13–18 mo had lower *T* scores of emotionally reactive behavior, somatic complaints, attention problems, aggressive behavior, as well as DSM-V-oriented pervasive developmental problems, attention deficit/hyperactivity problems, and oppositional defiant problems (Table 4). Multivariable logistic regression models showed that breastfeeding for periods of 7–12 mo, 13–18 mo, and >18 mo was associated with lower risks of externalizing problems [a*OR* (95% *CI*): 0.96 (0.92, 0.99), 0.94 (0.91, 0.98), 0.96 (0.92, 0.99), respectively], and breastfeeding for 13–18 mo was associated with a lower risk of total problems (a*OR*: 0.95, 95% *CI*: 0.90, 0.99) compared with breastfeeding for ≤6 mo (Figure 2). No other behavioral or emotional scales were significantly associated with the duration of breastfeeding.

### 3.4. Subgroup Analysis Based on Children’s Sex

When we performed a subgroup analysis stratified by children’s sex, the significant associations of feeding patterns and breastfeeding duration with behavioral and emotional scales remained in boys but not in girls. In boys, compared with non-EBF children, those who underwent EBF had lower *T* scores for almost all behavioral and emotional scales except for sleep problems, stress problems, and DSM-V-oriented pervasive developmental problems (Table A3), and they had significantly lower risks of clinically internalizing problems, externalizing problems, total problems, as well as DSM-V-oriented attention deficit/hyperactivity problems (Table A4). As for the duration of breastfeeding, boys who were breastfed for 13–18 mo had lower *T* scores for internalizing problems, externalizing problems, total problems, emotionally reactive behavior, anxiety/depression, withdrawn behavior, attention problems, aggressive behavior, as well as DSM-V-oriented pervasive developmental problems, attention deficit/hyperactivity problems, and oppositional defiant problems (Table A5); they also had lower risks of clinically externalizing problems (Table A6).

## 4. Discussion

In this prospective cohort study among 2489 children from China, we found that breastfeeding practices including EBF for the first 6 mo of life and a prolonged duration of breastfeeding were significantly associated with better behavioral and emotional performance in children at ages in the range of 1.5–5.0 y. We also found that these beneficial associations were more likely exhibited in boys rather than in girls.

In our population, children who underwent EBF for the first 6 mo of life versus non-EBF children had lower *T* scores for behavioral and emotional problems and lower risks of the corresponding clinical problems. These findings were supported by a previous American study which examined the combined effects of EBF and breastfeeding duration on psychosocial development using the Strengths and Difficulties Questionnaire, a briefer tool in assessing behavioral performance. This study showed that ≥3 mo of EBF combined with ≥6 mo of breastfeeding versus non-breastfeeding was associated with better emotional and conduct performance in children at 6 y old [16]. Another cohort study in Chinese children aged 5 y also found that EBF children had a lower score of emotional problems assessed by CBCL compared to non-EBF children [17]. It is worth noting that the differences in symptoms or composite scores might not be clinically meaningful, but they are relevant on a population level [32].

Our study showed that a longer duration of breastfeeding was associated with better childhood behavioral and emotional development outcomes, whether measured continuously or categorically. These results were consistent with findings from most previous studies that examined the effect of breastfeeding duration within 12 mo, which reported that a longer duration of breastfeeding could improve behavioral and emotional performance in children [17,19,20,21,24,25,33]. When associations were observed, a duration of 4–6 mo appeared to be most common, and two studies extended the durations to 9 and 10 months. Our study extended the duration of breastfeeding to beyond 18 mo and found that the risks of total problems were significantly reduced in children who were breastfed for 13–18 mo versus those who were breastfed for ≤6 mo. Our study also extended previous work on the non-linear relationships between the duration of breastfeeding and the *T* scores of child behavioral and emotional problems. Inflection points were observed around 6 mo and 18 mo, and the *T* scores significantly decreased during the 6–18 mo period for both behavioral and emotional problems. These results support the WHO recommendations for breastfeeding children up to 2 y of age or beyond.

Several potential mechanisms may explain the links between breastfeeding practice and child behavioral and emotional development. First, breast milk contains large amounts of essential long chain polyunsaturated fatty acids (LCPUFAs), such as docosahexaenoic acid (DHA), which are essential for central nervous system development and may improve cognitive scores [34]. Second, the milk fat globule membrane (MFGM), a heterogeneous mixture of proteins, phospholipids, sphingolipids, gangliosides, choline, and sialic acid, improves neurodevelopment in cognitive, mental, and motor outcomes [35]. Third, non-nutrient bioactive factors in breast milk, such as human milk oligosaccharides (HMOs), lactoferrin, and microbes, were proven to have effects in early brain development [36,37]. These links could also be explained by mother–infant interaction during breastfeeding, which has been shown to benefit children’s behavioral outcomes through active talking, eye contact, and skin-to-skin touch [17]. As suggested by the differential susceptibility hypothesis, boys might be more sensitive to the aforementioned nutrient or non-nutrient exposure in early life, and more pronounced benefits of breastfeeding regarding behavioral and emotional development were exhibited [38], as shown in our subgroup analysis and the results from African children [39].

Our study also has some limitations. First, information on potential confounders, such as complementary feeding and formula types, was not collected in this study. Second, although the mothers generally had good mental health at recruitment, we did not collect data on maternal mental health, like postpartum anxiety, which might affect both feeding practices and children’s behavioral and emotional outcomes. Third, this study was conducted among a rural population, which limits the generalizability of the findings. Additionally, given the cohort study design, our findings might be influenced by residual confounding from unmeasured factors. Future studies considering mother–infant interaction are warranted to better understand the relationship between feeding practice and childhood neurobehavioral development.

## 5. Conclusions

In conclusion, we found that EBF for the first 6 mo of life and a prolonged duration of breastfeeding, exclusively or partially, to 13–18 mo benefits childhood behavioral and emotional performance. Our findings support the WHO recommendation on breastfeeding up to 2 y and highlight the necessity of strengthening breastfeeding in China, where less than 40% of infants are exclusively breastfed for the first 6 mo of life [40]. Further studies across regions and among different populations with a stricter control of bias are warranted to confirm these associations and explore the underlying mechanisms.

## Figures and Tables

**Table 1:** Maternal and children characteristics according to feeding patterns for first 6 mo of life.

Characteristics	Overall	Non-EBF	EBF	*p*
	(*N* = 2489)	(*N* = 404)	(*N* = 2085)	
Maternal				
Age at delivery, y, median (IQR)	22.7 (3.0)	22.6 (2.9)	22.7 (3.1)	0.081
Gestational age, wk, mean (SD)	39.7 (1.6)	39.6 (1.7)	39.7 (1.5)	0.165
Ethnicity, no. (%)				0.624
Han	2458 (98.8)	398 (98.5)	2060 (98.8)	
Other	31 (1.2)	6 (1.5)	25 (1.2)	
Education, no. (%)				0.116
≥High school	397 (16.0)	78 (19.3)	319 (15.3)	
Middle school	2066 (83.0)	321 (79.5)	1745 (83.7)	
≤Primary school	26 (1.0)	5 (1.2)	21 (1.0)	
Occupation, no. (%)				<0.001
Farmer	2327 (93.5)	358 (88.6)	1969 (94.4)	
Other	162 (6.5)	46 (11.4)	116 (5.6)	
BMI in early pregnancy, kg/m^2^, no. (%)				0.014
<18.5	207 (8.3)	49 (12.1)	158 (7.6)	
18.5–22.9	1562 (62.8)	237 (58.7)	1325 (63.5)	
23.0–27.4	612 (24.6)	97 (24.0)	515 (24.7)	
≥27.5	108 (4.3)	21 (5.2)	87 (4.2)	
Anemia in mid-pregnancy, no. (%)				0.359
No	2329 (93.6)	372 (92.1)	1957 (93.9)	
Yes	149 (6.0)	28 (6.9)	121 (5.8)	
Missing	11(0.4)	4(1.0)	7(0.3)	
Supplementation during pregnancy, no. (%)				0.937
Folic acid	812 (32.6)	129 (31.9)	683 (32.8)	
Iron–folic acid	855 (34.4)	139 (34.4)	716 (34.3)	
Multiple micronutrients	822 (33.0)	136 (33.7)	686 (32.9)	
Delivery mode, no. (%)				0.624
Vaginal delivery	1300 (52.2)	216 (53.5)	1084 (52.0)	
Cesarean delivery	1188 (47.7)	188 (46.5)	1000 (48.0)	
Missing	1(0.0)	0(0)	1(0.0)	
Children				
Sex, no. (%)				0.014
Male	1290 (51.8)	232 (57.4)	1058 (50.7)	
Female	1199 (48.2)	172 (42.6)	1027 (49.3)	
Birthweight, no. (%)				0.354
<2500	43 (1.7)	9 (2.2)	34 (1.6)	
2500–3999	2337 (93.9)	373 (92.3)	1964 (94.2)	
≥4000	109 (4.4)	22 (5.4)	87 (4.2)	
Age at follow-up, mo, median (IQR)	32.0 (17.0)	31.0 (14.1)	32.0 (17.0)	0.172
Age at follow-up, mo, no. (%)				0.051
18–29	1102 (44.3)	178 (44.1)	924 (44.3)	
30–35	437 (17.6)	90 (22.3)	347 (16.6)	
36–41	280 (11.2)	44 (10.9)	236 (11.3)	
42–47	400 (16.1)	53 (13.1)	347 (16.6)	
48–60	270 (10.8)	39 (9.7)	231 (11.1)	
Main caregivers, no. (%)				<0.001
Parents	1975 (79.3)	265 (65.6)	1710 (82.0)	
Grandparents	510 (20.5)	139 (34.4)	371 (17.8)	
Missing	4 (0.2)	0 (0)	4 (0.2)	
Kindergarten attendance, no. (%)				0.035
Yes	1835 (73.7)	281 (69.6)	1554 (74.5)	
No	650 (26.1)	123 (30.4)	527 (25.3)	
Missing	4 (0.2)	0 (0)	4 (0.2)	

**Table 2:** Mean differences in *T* scores for child behavioral and emotional problems based on feeding patterns for first 6 mo of life; mean (SD).

Items	Non-EBF (*n* = 404)	EBF (*n* = 2085)	cMD (95% *CI*)	*p*	aMD (95% *CI*) *	*p*
Syndrome						
Emotionally reactive	54.4 (5.78)	53.9 (5.71)	−0.48 (−1.09, 0.13)	0.124	−0.49 (−1.11, 0.14)	0.126
Anxious/depressed	53.6 (5.35)	53.3 (5.49)	−0.35 (−0.93, 0.23)	0.241	−0.46 (−1.06, 0.13)	0.126
Somatic complaints	55.2 (6.67)	53.9 (6.07)	−1.25 (−1.91, −0.60)	<0.001	−1.26 (−1.93, −0.59)	<0.001
Withdrawn behavior	55.7 (6.47)	55.2 (6.69)	−0.50 (−1.21, 0.21)	0.166	−0.37 (−1.09, 0.36)	0.320
Sleep problems	52.3 (3.94)	52.2 (3.92)	−0.13 (−0.55, 0.29)	0.536	−0.14 (−0.57, 0.28)	0.511
Attention problems	53.8 (5.19)	53.2 (4.79)	−0.53 (−1.05, −0.01)	0.045	−0.26 (−0.79, 0.26)	0.324
Aggressive behavior	53.0 (5.17)	52.3 (4.63)	−0.74 (−1.24, −0.23)	0.004	−0.57 (−1.08, −0.06)	0.030
Stress problems	53.8 (5.64)	53.3 (5.53)	−0.47 (−1.06, 0.12)	0.121	−0.48 (−1.08, 0.13)	0.122
Composite problem						
Internalizing problems	50.1 (11.4)	48.7 (11.2)	−1.39 (−2.59, −0.20)	0.022	−1.31 (−2.53, −0.10)	0.034
Externalizing problems	47.6 (10.1)	46.3 (9.56)	−1.33 (−2.36, −0.30)	0.011	−0.88 (−1.92, 0.15)	0.094
Total problems	48.6 (11.5)	47.0 (10.9)	−1.54 (−2.71, −0.37)	0.010	−1.26 (−2.44, −0.08)	0.037
DSM-oriented problem						
Affective problems	55.6 (6.96)	55.1 (6.60)	−0.53 (−1.24, 0.18)	0.140	−0.54 (−1.26, 0.18)	0.142
Anxiety problems	54.9 (6.85)	54.2 (6.17)	−0.66 (−1.33, 0.01)	0.053	−0.79 (−1.47, −0.10)	0.024
Pervasive developmental problems	55.6 (6.92)	55.1 (7.07)	−0.49 (−1.24, 0.26)	0.204	−0.38 (−1.14, 0.39)	0.336
Attention deficit/hyperactivity problems	54.1 (5.63)	53.4 (5.03)	−0.69 (−1.23, −0.14)	0.014	−0.45 (−1.00, 0.11)	0.114
Oppositional defiant problems	52.4 (4.35)	52.0 (4.12)	−0.46 (−0.90, −0.02)	0.042	−0.34 (−0.79, 0.11)	0.141

Notes: * Adjusted for maternal age, gestational week, education, occupation, BMI in early pregnancy, anemia in mid-pregnancy, supplementation during pregnancy, delivery mode, children’s sex, age, birthweight, main caregiver, and kindergarten attendance.

**Table 3:** Risks of childhood behavioral and emotional problems based on feeding patterns for first 6 mo of life.

Items	Non-EBF (*n* = 404)	EBF (*n* = 2085)				
	Events (%)	Events (%)	c*OR* (95% *CI*)	*p*	a*OR* (95% *CI*) *	*p*
Syndrome						
Emotionally reactive	5 (1.2)	25 (1.2)	0.97 (0.37, 2.54)	0.948	0.87 (0.32, 2.34)	0.785
Anxious/depressed	6 (1.5)	37 (1.8)	1.20 (0.50, 2.86)	0.683	1.12 (0.46, 2.72)	0.802
Somatic complaints	19 (4.7)	66 (3.2)	0.66 (0.39, 1.12)	0.122	0.67 (0.39, 1.15)	0.144
Withdrawn behavior	20 (5.0)	103 (4.9)	1.00 (0.61, 1.63)	0.993	1.04 (0.63, 1.72)	0.889
Sleep problems	2 (0.5)	14 (0.7)	1.36 (0.31, 6.00)	0.686	1.29 (0.28, 5.90)	0.741
Attention problems	8 (2.0)	35 (1.7)	0.85 (0.39, 1.84)	0.671	0.95 (0.43, 2.10)	0.891
Aggressive behavior	3 (0.7)	21 (1.0)	1.36 (0.40, 4.58)	0.620	1.51 (0.43, 5.28)	0.521
Stress problems	11 (2.7)	63 (3.0)	1.11 (0.58, 2.13)	0.746	1.11 (0.57, 2.16)	0.752
Composite problem						
Internalizing problems	49 (12.1)	200 (9.6)	0.77 (0.55, 1.07)	0.121	0.75 (0.54, 1.06)	0.106
Externalizing problems	27 (6.7)	72 (3.5)	0.50 (0.32, 0.79)	0.003	0.54 (0.34, 0.87)	0.011
Total problems	41 (10.1)	147 (7.1)	0.67 (0.47, 0.97)	0.032	0.69 (0.47, 1.01)	0.056
DSM-oriented problem						
Affective problems	29 (7.2)	122 (5.9)	0.80 (0.53, 1.22)	0.307	0.77 (0.50, 1.18)	0.230
Anxiety problems	21 (5.2)	83 (4.0)	0.76 (0.46, 1.24)	0.265	0.70 (0.42, 1.16)	0.164
Pervasive developmental problems	27 (6.7)	129 (6.2)	0.92 (0.60, 1.41)	0.707	0.89 (0.58, 1.39)	0.620
Attention deficit/hyperactivity problems	15 (3.7)	26 (1.2)	0.33 (0.17, 0.62)	0.001	0.35 (0.18, 0.69)	0.002
Oppositional defiant problems	5 (1.2)	36 (1.7)	1.40 (0.55, 3.59)	0.482	1.64 (0.62, 4.30)	0.317

Notes: * Adjusted for maternal age, gestational week, education, occupation, BMI in early pregnancy, anemia in mid-pregnancy, supplementation during pregnancy, delivery mode, children’s sex, age, birthweight, main caregiver, and kindergarten attendance.

**Table 4:** Mean differences of *T* scores for child behavioral and emotional problems based on duration of breastfeeding.

Items			7–12 mo (*n* = 489)			13–18 mo (*n* = 1408)				>18 mo (*n* = 450)		
	cMD (95% *CI*)	*p*	aMD (95% *CI*) *	*p*	cMD (95% *CI*)	*p*	aMD (95% *CI*) *	*p*	cMD (95% *CI*)	*p*	aMD (95% *CI*) *	*p*
Syndrome												
Emotionally reactive	−0.47 (−1.54, 0.59)	0.385	−0.33 (−1.40, 0.74)	0.550	−1.29 (−2.28, −0.31)	0.010	−1.22 (−2.22, −0.23)	0.016	−0.82 (−1.89, 0.26)	0.138	−0.79 (−1.88, 0.29)	0.152
Anxious/depressed	0.24 (−0.78, 1.25)	0.650	0.32 (−0.70, 1.34)	0.537	−0.84 (−1.78, 0.10)	0.079	−0.92 (−1.87, 0.03)	0.058	−0.44 (−1.47, 0.59)	0.398	−0.53 (−1.57, 0.51)	0.317
Somatic complaints	0.07 (−1.08, 1.22)	0.907	0.13 (−1.03, 1.28)	0.832	−1.05 (−2.12, 0.01)	0.053	−1.08 (−2.16, −0.01)	0.048	−0.84 (−2.01, 0.32)	0.157	−0.88 (−2.05, 0.30)	0.144
Withdrawn behavior	0.11 (−1.13, 1.35)	0.861	0.24 (−1.00, 1.49)	0.703	−1.05 (−2.20, 0.10)	0.073	−0.88 (−2.03, 0.28)	0.138	−0.42 (−1.67, 0.84)	0.515	−0.27 (−1.53, 0.99)	0.674
Sleep problems	0.08 (−0.65, 0.82)	0.821	0.18 (−0.55, 0.92)	0.629	−0.15 (−0.83, 0.52)	0.658	−0.12 (−0.80, 0.56)	0.726	−0.03 (−0.77, 0.71)	0.943	−0.01 (−0.76, 0.73)	0.976
Attention problems	−0.53 (−1.44, 0.37)	0.249	−0.37 (−1.27, 0.53)	0.423	−1.27 (−2.11, −0.44)	0.003	−1.01 (−1.85, −0.18)	0.017	−0.97 (−1.88, −0.05)	0.038	−0.78 (−1.69, 0.13)	0.095
Aggressive behavior	−0.71 (−1.59, 0.17)	0.113	−0.53 (−1.41, 0.35)	0.238	−1.60 (−2.41, −0.79)	<0.001	−1.42 (−2.23, −0.60)	0.001	−1.19 (−2.08, −0.30)	0.009	−1.05 (−1.94, −0.16)	0.021
Stress problems	0.30 (−0.74, 1.33)	0.573	0.34 (−0.70, 1.38)	0.523	−0.62 (−1.57, 0.34)	0.205	−0.62 (−1.58, 0.35)	0.210	−0.42 (−1.47, 0.62)	0.426	−0.48 (−1.53, 0.58)	0.377
Composite problem												
Internalizing problems	−0.29 (−2.38, 1.80)	0.788	0.04 (−2.05, 2.13)	0.971	−2.68 (−4.61, −0.75)	0.007	−2.50 (−4.43, −0.56)	0.012	−1.15 (−3.26, 0.96)	0.285	−1.07 (−3.19, 1.04)	0.320
Externalizing problems	−1.47 (−3.27, 0.33)	0.110	−1.02 (−2.79, 0.76)	0.262	−3.28 (−4.94, −1.61)	<0.001	−2.75 (−4.40, −1.10)	0.001	−2.28 (−4.09, −0.46)	0.014	−1.88 (−3.68, −0.08)	0.041
Total problems	−1.16 (−3.20, 0.89)	0.268	−0.72 (−2.74, 1.31)	0.488	−3.32 (−5.21, −1.44)	0.001	−2.96 (−4.84, −1.07)	0.002	−2.01 (−4.08, 0.05)	0.056	−1.78 (−3.84, 0.28)	0.090
DSM-oriented problem												
Affective problems	−0.03 (−1.28, 1.21)	0.957	0.06 (−1.18, 1.30)	0.926	−0.96 (−2.11, 0.18)	0.100	−0.97 (−2.12, 0.19)	0.102	−0.20 (−1.46, 1.05)	0.751	−0.25 (−1.51, 1.02)	0.701
Anxiety problems	−0.04 (−1.22, 1.13)	0.816	−0.04 (−1.22, 1.13)	0.941	−1.19 (−2.29, −0.10)	0.039	−1.19 (−2.29, −0.10)	0.032	−0.36 (−1.56, 0.83)	0.631	−0.36 (−1.56, 0.83)	0.551
Pervasive developmental problems	−0.38 (−1.70, 0.93)	0.409	−0.55 (−1.87, 0.76)	0.569	−1.24 (−2.46, −0.02)	0.030	−1.34 (−2.56, −0.13)	0.047	−0.49 (−1.83, 0.84)	0.381	−0.59 (−1.92, 0.73)	0.469
Attention deficit/hyperactivity problems	−0.82 (−1.78, 0.14)	0.093	−0.66 (−1.61, 0.29)	0.175	−1.55 (−2.43, −0.66)	0.001	−1.29 (−2.17, −0.41)	0.004	−1.05 (−2.02, −0.09)	0.033	−0.88 (−1.85, 0.08)	0.074
Oppositional defiant problems	−0.01 (−0.79, 0.76)	0.97	0.07 (−0.71, 0.84)	0.863	−1.05 (−1.76, −0.33)	0.004	−0.93 (−1.65, −0.21)	0.011	−0.49 (−1.27, 0.29)	0.222	−0.39 (−1.18, 0.40)	0.333

Notes: * Adjusted for maternal age, gestational week, education, occupation, BMI in early pregnancy, anemia in mid-pregnancy, supplementation during pregnancy, delivery mode, children’s sex, age, birthweight, main caregiver, and kindergarten attendance.

## Data Availability

The data are presented in the manuscript; the code book and analytic code will not be made available because specific consent has not been obtained from the participants nor the Ethics Committee when receiving approval for this study at the time it was conducted.

## Appendix A

**Table A1:** Characteristics of excluded and included populations.

Characteristics	Overall	Included	Excluded
	(*N* = 17,613)	(*N* = 2489)	(*N* = 15,124)
Maternal			
Age, y, median (IQR)	22.9 (3.20)	22.7 (3.00)	23.0 (3.20)
Gestational week, wk, mean (SD)	39.6 (1.67)	39.7 (1.57)	39.6 (1.68)
Education, no. (%)			
≥High school	3204 (18.2)	397 (16.0)	2807 (18.6)
Middle school	14,130 (80.2)	2066 (83.0)	12,064 (79.8)
≤Primary school	279 (11.6)	26 (1.0)	253 (1.7)
Occupation, no. (%)			
Farmer	16,018 (90.9)	2327 (93.5)	13,691 (90.5)
Others	1595 (9.1)	162 (6.5)	1433 (9.5)
BMI in early pregnancy, kg/m^2^, no. (%)			
<18.5	1568 (8.9)	207 (8.3)	1361 (9.0)
18.5–22.9	11,115 (63.1)	1562 (62.8)	9553 (63.2)
23.0–27.4	4187 (23.8)	612 (24.6)	3575 (23.6)
≥27.5	743 (4.2)	108 (4.3)	635 (4.2)
Anemia in mid-pregnancy, no. (%)			
Yes	16,402 (93.2)	2329 (93.6)	14,073 (93.1)
No	1097 (6.2)	149 (6.0)	948 (6.3)
Missing	114 (0.6)	11 (0.4)	103 (0.7)
Supplementation during pregnancy, no. (%)			
Folic acid	5861 (33.3)	812 (32.6)	5049 (33.4)
Iron–folic acid	5882 (33.4)	855 (34.4)	5027 (33.2)
Multiple micronutrients	5870 (33.3)	822 (33.0)	5048 (33.4)
Delivery mode, no. (%)			
Vaginal delivery	8995 (51.1)	1300 (52.2)	7695 (50.9)
Cesarean delivery	8545 (48.5)	1188 (47.7)	7357 (48.6)
Missing	73 (0.4)	1 (0.0)	72 (0.5)
Offspring			
Age, mo, median (IQR)	38.0 (1.00)	39.0 (1.00)	38.0 (1.00)
Sex, no. (%)			
Male	9240 (52.5)	1290 (51.8)	7950 (52.6)
Female	8365 (47.5)	1199 (48.2)	7166 (47.4)
Missing	8 (0.0)	0 (0)	8 (0.1)
Birthweight, g, mean (SD)	3300 (386)	3290 (377)	3300 (388)
Feeding pattern for the first 6 mo, no. (%)			
Exclusive breastfeeding	12,342 (70.1)	2085 (83.8)	10,257 (67.2)
Non-exclusive breastfeeding	2781 (15.8)	404 (16.2)	2377 (15.6)
Missing	2625 (14.9)	0 (0)	2625 (17.2)
Duration of breastfeeding, mo, mean (SD)	15.3 (5.26)	15.5 (5.09)	15.3 (5.30)
Main caregivers, no. (%)			
Parents	11,489 (65.2)	1975 (79.3)	9514 (62.9)
Grandparents	3595 (20.4)	510 (20.5)	3085 (20.4)
Missing	2529 (14.4)	4 (0.2)	2525 (16.7)
Kindergarten attendance, no. (%)			
Yes	8902 (50.5)	1835 (73.7)	7067 (46.7)
No	6207 (35.2)	650 (26.1)	5557 (36.7)
Missing	2504 (14.2)	4 (0.2)	2500 (16.5)

**Table A2:** Segmented linear regression for association between duration of breastfeeding and *T* scores for internalizing problems, externalizing problems, and total problems.

Items	≤6 mo		7–18 mo		>18 mo	
	*βadj* (95% *CI*)	*p*	*βadj* (95% *CI*)	*p*	*βadj* (95% *CI*)	*p*
Syndrome						
Emotionally reactive	0.05 (−0.38, 0.49)	0.813	−0.14 (−0.23, −0.05)	0.003	0.01 (−0.19, 0.21)	0.927
Anxious/depressed	0.24 (−0.19, 0.66)	0.274	−0.16 (−0.25, −0.08)	<0.001	0.04 (−0.14, 0.21)	0.664
Somatic complaints	−0.08 (−0.56, 0.40)	0.750	−0.14 (−0.24, −0.04)	0.005	0.14 (−0.05, 0.33)	0.153
Withdrawn behavior	0.20 (−0.25, 0.64)	0.390	−0.19 (−0.30, −0.09)	<0.001	0.18 (−0.03, 0.39)	0.099
Sleep problems	0.01 (−0.31, 0.33)	0.957	−0.04 (−0.10, 0.02)	0.228	−0.02 (−0.14, 0.10)	0.718
Attention problems	0.02 (−0.36, 0.39)	0.929	−0.06 (−0.14, 0.02)	0.122	0.04 (−0.13, 0.20)	0.674
Aggressive behavior	−0.15 (−0.56, 0.27)	0.494	−0.11 (−0.19, −0.04)	0.002	0.12 (−0.03, 0.28)	0.125
Stress problems	−0.07 (−0.47, 0.33)	0.728	−0.13 (−0.22, −0.05)	0.003	0.06 (−0.12, 0.24)	0.532
Composite problem						
Internalizing problems	0.41 (−0.40, 1.22)	0.321	−0.39 (−0.57, −0.21)	<0.001	0.19 (−0.17, 0.55)	0.303
Externalizing problems	0.02 (−0.69, 0.72)	0.960	−0.27 (−0.42, −0.12)	<0.001	0.15 (−0.16, 0.47)	0.333
Total problems	0.21 (−0.60, 1.03)	0.610	−0.33 (−0.50, −0.15)	<0.001	0.19 (−0.17, 0.54)	0.302
DSM-oriented problem						
Affective problems	0.23 (−0.28, 0.74)	0.375	−0.14 (−0.25, −0.04)	0.007	0.14 (−0.08, 0.37)	0.216
Anxiety problems	−0.10 (−0.61, 0.41)	0.713	−0.17 (−0.27, −0.07)	0.001	0.02 (−0.19, 0.23)	0.866
Pervasive developmental problems	0.02 (−0.50, 0.54)	0.948	−0.13 (−0.24, −0.02)	0.023	0.23 (0.01, 0.46)	0.045
Attention deficit/hyperactivity problems	−0.22 (−0.62, 0.18)	0.279	−0.03 (−0.11, 0.05)	0.504	−0.05 (−0.22, 0.11)	0.529
Oppositional defiant problems	−0.04 (−0.36, 0.28)	0.824	−0.15 (−0.21, −0.08)	<0.001	0.11 (−0.04, 0.27)	0.146

Notes: Negative values indicate decrease in *T* score of behavioral and emotional problems, which means better behavioral and emotional development; positive values indicate increase in *T* score of behavioral and emotional problems, which means worse behavioral and emotional development. All models were adjusted for maternal age, gestational week, education, occupation, BMI in early pregnancy, anemia in mid-pregnancy, supplementation during pregnancy, delivery mode, children’s sex, age, birthweight, main caregiver, and kindergarten attendance.

**Table A3:** Mean differences in *T* scores for childhood behavioral and emotional problems based on feeding pattern (EBF vs. non-EBF) for first 6 mo of life stratified by children’s sex.

Items	Boys (*n* = 1290)		Girls (*n* = 1199)		*p* for Interaction
	aMD (95% *CI*)	*p*	aMD (95% *CI*)	*p*	
Syndrome					
Emotionally reactive	−1.01 (−1.81, −0.20)	0.014	0.19 (−0.79, 1.16)	0.707	0.093
Anxious/depressed	−1.04 (−1.82, −0.26)	0.009	0.25 (−0.68, 1.17)	0.602	0.029
Somatic complaints	−1.03 (−1.95, −0.11)	0.028	−1.53 (−2.52, −0.53)	0.003	0.479
Withdrawn behavior	−1.14 (−2.08, −0.20)	0.018	0.70 (−0.42, 1.83)	0.220	0.009
Sleep problems	−0.25 (−0.78, 0.29)	0.365	0.05 (−0.64, 0.74)	0.884	0.458
Attention problems	−0.85 (−1.56, −0.14)	0.019	0.51 (−0.27, 1.28)	0.198	0.011
Aggressive behavior	−1.20 (−1.92, −0.47)	0.001	0.24 (−0.48, 0.95)	0.518	0.009
Stress problems	−0.71 (−1.51, 0.09)	0.083	−0.15 (−1.08, 0.77)	0.749	0.423
Composite problem					
Internalizing problems	−2.12 (−3.75, −0.50)	0.011	−0.16 (−2.00, 1.67)	0.862	0.115
Externalizing problems	−1.87 (−3.29, −0.46)	0.010	0.41 (−1.11, 1.93)	0.599	0.048
Total problems	−2.24 (−3.83, −0.65)	0.006	0.11 (−1.66, 1.88)	0.902	0.056
DSM-oriented problem					
Affective problems	−0.97 (−1.91, −0.03)	0.043	0.08 (−1.05, 1.20)	0.894	0.080
Anxiety problems	−1.24 (−2.13, −0.34)	0.007	−0.20 (−1.26, 0.86)	0.711	0.149
Pervasive developmental problems	−0.99 (−2.00, 0.01)	0.053	0.40 (−0.78, 1.58)	0.509	0.071
Attention deficit/hyperactivity problems	−0.92 (−1.69, −0.15)	0.019	0.15 (−0.64, 0.95)	0.706	0.048
Oppositional defiant problems	−0.72 (−1.36, −0.07)	0.029	0.15 (−0.48, 0.79)	0.636	0.071

Notes: Adjusted for maternal age, gestational week, education, occupation, BMI in early pregnancy, anemia in mid-pregnancy, supplementation during pregnancy, delivery mode, children’s sex, age, birthweight, main caregiver, and kindergarten attendance. *p* for interaction was *p*-value of interaction term for sex and feeding pattern for first 6 mo.

**Table A4:** Risks of child behavioral and emotional problems based on feeding pattern (EBF vs. non-EBF) for first 6 mo stratified by children’s sex.

Items	Boys (*n* = 1290)		Girls (*n* = 1199)	
	a*OR* (95% *CI*)	*p*	a*OR* (95% *CI*)	*p*
Syndrome				
Emotionally reactive	0.67 (0.17, 2.58)	0.558	1.26 (0.27, 5.86)	0.772
Anxious/depressed	1.19 (0.33, 4.35)	0.788	1.09 (0.31, 3.92)	0.890
Somatic complaints	0.75 (0.37, 1.55)	0.442	0.52 (0.22, 1.19)	0.122
Withdrawn behavior	0.81 (0.42, 1.59)	0.544	1.58 (0.70, 3.60)	0.271
Sleep problems	/	0.997	0.72 (0.14, 3.66)	0.695
Attention problems	0.53 (0.19, 1.44)	0.210	2.02 (0.45, 9.08)	0.361
Aggressive behavior	1.61 (0.35, 7.45)	0.542	2.04 (0.17, 24.52)	0.572
Stress problems	1.40 (0.52, 3.75)	0.503	0.95 (0.38, 2.38)	0.918
Composite problem				
Internalizing problems	0.62 (0.39, 0.97)	0.037	0.98 (0.57, 1.70)	0.956
Externalizing problems	0.40 (0.23, 0.71)	0.002	1.07 (0.40, 2.87)	0.895
Total problems	0.54 (0.33, 0.86)	0.011	1.02 (0.53, 1.95)	0.958
DSM-oriented problem				
Affective problems	0.67 (0.38, 1.20)	0.180	0.98 (0.50, 1.94)	0.951
Anxiety problems	0.60 (0.30, 1.19)	0.145	0.82 (0.39, 1.75)	0.610
Pervasive developmental problems	0.79 (0.44, 1.43)	0.432	1.01 (0.51, 1.98)	0.980
Attention deficit/hyperactivity problems	0.34 (0.14, 0.80)	0.014	0.46 (0.14, 1.46)	0.187
Oppositional defiant problems	1.44 (0.47, 4.42)	0.525	2.50 (0.31, 19.90)	0.388

Notes: Adjusted for maternal age, gestational week, education, occupation, BMI in early pregnancy, anemia in mid-pregnancy, supplementation during pregnancy, delivery mode, children’s sex, age, birthweight, main caregiver, and kindergarten attendance.

**Table A5:** Mean differences in *T* scores for childhood behavioral and emotional problems based on duration of breastfeeding, stratified by children’s sex, aMD (95% *CI*).

Items			Boys (*n* = 1290)							Girls (*n* = 1199)		
	7–12 mo	*p*	13–18 mo	*p*	>18 mo	*p*	7–12 mo	*p*	13–18 mo	*p*	>18 mo	*p*
Syndrome												
Emotionally reactive	−0.58 (−1.97, 0.80)	0.411	−1.58 (−2.85, −0.31)	0.015	−0.88 (−2.27, 0.52)	0.218	−0.01 (−1.68, 1.66)	0.990	−0.70 (−2.27, 0.88)	0.387	−0.57 (−2.27, 1.14)	0.515
Anxious/depressed	−0.14 (−1.48, 1.20)	0.838	−1.75 (−2.97, −0.52)	0.005	−1.07 (−2.42, 0.27)	0.119	0.93 (−0.65, 2.51)	0.250	0.16 (−1.33, 1.65)	0.832	0.24 (−1.37, 1.85)	0.769
Somatic complaints	0.47 (−1.11, 2.06)	0.558	−1.38 (−2.83, 0.07)	0.063	−0.80 (−2.39, 0.80)	0.326	−0.12 (−1.83, 1.60)	0.895	−0.66 (−2.28, 0.96)	0.424	−0.82 (−2.57, 0.93)	0.358
Withdrawn behavior	−0.43 (−2.06, 1.20)	0.607	−1.67 (−3.16, −0.18)	0.028	−0.59 (−2.23, 1.05)	0.484	1.09 (−0.84, 3.02)	0.269	0.25 (−1.57, 2.06)	0.791	0.22 (−1.75, 2.18)	0.830
Sleep problems	0.23 (−0.69, 1.16)	0.621	−0.12 (−0.97, 0.73)	0.785	−0.04 (−0.97, 0.90)	0.939	0.16 (−1.02, 1.34)	0.789	−0.08 (−1.19, 1.03)	0.893	0.09 (−1.12, 1.29)	0.889
Attention problems	−0.48 (−1.71, 0.74)	0.440	−1.49 (−2.61, −0.36)	0.010	−1.13 (−2.36, 0.11)	0.074	−0.17 (−1.50, 1.16)	0.801	−0.35 (−1.61, 0.90)	0.580	−0.31 (−1.67, 1.05)	0.655
Aggressive behavior	−0.54 (−1.79, 0.71)	0.397	−1.89 (−3.03, −0.74)	0.001	−1.15 (−2.41, 0.11)	0.074	−0.51 (−1.73, 0.72)	0.416	−0.82 (−1.97, 0.34)	0.165	−0.89 (−2.14, 0.36)	0.161
Stress problems	0.40 (−0.98, 1.78)	0.573	−1.02 (−2.28, 0.25)	0.115	−0.40 (−1.79, 0.99)	0.574	0.37 (−1.22, 1.95)	0.649	−0.06 (−1.56, 1.43)	0.934	−0.46 (−2.07, 1.16)	0.581
Composite problem												
Internalizing problems	−0.43 (−3.23, 2.37)	0.766	−3.48 (−6.05, −0.92)	0.008	−1.31 (−4.13, 1.51)	0.361	0.76 (−2.38, 3.91)	0.636	−1.04 (−4.01, 1.92)	0.490	−0.54 (−3.75, 2.67)	0.742
Externalizing problems	−0.76 (−3.20, 1.68)	0.544	−3.26 (−5.50, −1.03)	0.004	−1.63 (−4.09, 0.83)	0.194	−1.29 (−3.89, 1.32)	0.334	−2.09 (−4.54, 0.37)	0.097	−2.16 (−4.82, 0.50)	0.112
Total problems	−0.65 (−3.39, 2.09)	0.642	−3.57 (−6.08, −1.06)	0.005	−1.76 (−4.52, 1.00)	0.212	−0.67 (−3.70, 2.36)	0.666	−2.04 (−4.89, 0.82)	0.162	−1.67 (−4.76, 1.42)	0.289
DSM-oriented problem												
Affective problems	0.07 (−1.56, 1.69)	0.937	−1.41 (−2.90, 0.08)	0.063	−0.26 (−1.89, 1.38)	0.760	0.15 (−1.78, 2.08)	0.882	−0.33 (−2.15, 1.49)	0.722	−0.07 (−2.04, 1.90)	0.944
Anxiety problems	−0.86 (−2.41, 0.68)	0.272	−1.99 (−3.40, −0.58)	0.006	−1.12 (−2.67, 0.44)	0.159	0.98 (−0.84, 2.80)	0.291	−0.08 (−1.79, 1.64)	0.930	0.65 (−1.20, 2.51)	0.490
Pervasive developmental problems	−1.24 (−2.98, 0.51)	0.180	−2.02 (−3.61, −0.44)	0.015	−0.85 (−2.59, 0.90)	0.417	0.31 (−1.69, 2.32)	0.627	−0.47 (−2.34, 1.41)	0.779	−0.19 (−2.24, 1.85)	0.892
Attention deficit/hyperactivity problems	−0.35 (−1.69, 0.98)	0.604	−1.44 (−2.67, −0.22)	0.021	−0.96 (−2.31, 0.38)	0.160	−1.03 (−2.39, 0.34)	0.140	−1.13 (−2.42, 0.16)	0.086	−0.82 (−2.22, 0.57)	0.246
Oppositional defiant problems	−0.11 (−1.22, 0.99)	0.840	−1.33 (−2.35, −0.32)	0.010	−0.52 (−1.64, 0.59)	0.357	0.27 (−0.82, 1.36)	0.626	−0.47 (−1.49, 0.55)	0.369	−0.22 (−1.33, 0.89)	0.700

Notes: Adjusted for maternal age, gestational week, education, occupation, BMI in early pregnancy, anemia in mid-pregnancy, supplementation during pregnancy, delivery mode, children’s sex, age, birthweight, main caregiver, and kindergarten attendance.

**Table A6:** Risks of childhood behavioral and emotional problems based on duration of breastfeeding, stratified by children’s sex, a*OR* (95% *CI*).

Items			Boys (*n* = 1290)							Girls (*n* = 1199)		
	7–12 mo	*p*	13–18 mo	*p*	>18 mo	*p*	7–12 mo	*p*	13–18 mo	*p*	>18 mo	*p*
Syndrome												
Emotionally reactive	1.01 (0.98, 1.04)	0.439	0.99 (0.97, 1.01)	0.419	1.01 (0.98, 1.03)	0.530	1.00 (0.97, 1.04)	0.940	1.00 (0.97, 1.03)	0.998	0.99 (0.96, 1.03)	0.693
Anxious/depressed	1.01 (0.98, 1.04)	0.421	0.98 (0.95, 1.01)	0.209	0.99 (0.96, 1.02)	0.401	1.01 (0.97, 1.05)	0.568	1.00 (0.96, 1.04)	0.996	1.01 (0.97, 1.05)	0.751
Somatic complaints	1.01 (0.96, 1.06)	0.818	0.98 (0.94, 1.02)	0.322	0.97 (0.93, 1.02)	0.247	0.99 (0.94, 1.04)	0.600	0.99 (0.94, 1.03)	0.537	0.97 (0.93, 1.02)	0.255
Withdrawn behavior	1.00 (0.95, 1.05)	0.890	0.98 (0.94, 1.03)	0.424	0.96 (0.91, 1.01)	0.136	1.01 (0.95, 1.08)	0.691	0.98 (0.93, 1.05)	0.621	0.99 (0.92, 1.05)	0.677
Sleep problems	1.01 (0.99, 1.02)	0.264	1.01 (0.99, 1.02)	0.448	1.00 (0.99, 1.02)	0.676	0.98 (0.95, 1.01)	0.158	0.97 (0.95, 1.00)	0.034	0.98 (0.95, 1.00)	0.077
Attention problems	1.00 (0.96, 1.03)	0.833	0.99 (0.96, 1.02)	0.454	0.99 (0.96, 1.03)	0.708	0.99 (0.95, 1.03)	0.626	0.99 (0.96, 1.03)	0.584	0.99 (0.96, 1.03)	0.741
Aggressive behavior	1.02 (0.99, 1.05)	0.207	0.99 (0.96, 1.01)	0.391	1.01 (0.98, 1.04)	0.405	0.99 (0.96, 1.01)	0.281	0.99 (0.97, 1.02)	0.569	0.99 (0.97, 1.02)	0.476
Stress problems	1.03 (0.98, 1.07)	0.233	0.99 (0.96, 1.03)	0.724	1.00 (0.96, 1.04)	0.992	0.98 (0.93, 1.03)	0.476	0.97 (0.92, 1.02)	0.202	0.96 (0.91, 1.01)	0.137
Composite problem												
Internalizing problems	0.97 (0.90, 1.04)	0.374	0.93 (0.87, 1.00)	0.039	0.94 (0.88, 1.02)	0.134	0.99 (0.91, 1.08)	0.829	0.96 (0.89, 1.04)	0.368	0.97 (0.89, 1.06)	0.542
Externalizing problems	0.94 (0.89, 1.00)	0.035	0.91 (0.87, 0.96)	<0.001	0.94 (0.89, 0.99)	0.031	0.97 (0.93, 1.02)	0.285	0.99 (0.94, 1.03)	0.519	0.98 (0.93, 1.03)	0.457
Total problems	1.00 (0.93, 1.07)	0.937	0.94 (0.88, 1.00)	0.038	0.96 (0.89, 1.03)	0.209	0.99 (0.92, 1.06)	0.786	0.96 (0.90, 1.03)	0.230	0.98 (0.91, 1.06)	0.678
DSM-oriented problem												
Affective problems	1.02 (0.96, 1.08)	0.497	0.98 (0.93, 1.03)	0.383	1.00 (0.94, 1.06)	0.974	0.98 (0.92, 1.05)	0.603	0.97 (0.91, 1.04)	0.399	1.00 (0.93, 1.07)	0.926
Anxiety problems	1.00 (0.95, 1.05)	0.959	0.96 (0.92, 1.01)	0.087	0.99 (0.94, 1.04)	0.575	1.02 (0.96, 1.08)	0.611	0.99 (0.94, 1.05)	0.793	1.00 (0.94, 1.06)	0.984
Pervasive developmental problems	0.98 (0.92, 1.04)	0.433	0.96 (0.91, 1.01)	0.148	0.98 (0.92, 1.04)	0.499	0.96 (0.89, 1.03)	0.214	0.94 (0.88, 1.00)	0.070	0.94 (0.87, 1.00)	0.067
Attention deficit/hyperactivity problems	1.02 (0.98, 1.05)	0.389	1.00 (0.97, 1.04)	0.799	1.00 (0.97, 1.04)	0.830	0.96 (0.93, 0.99)	0.007	0.95 (0.92, 0.97)	<0.001	0.94 (0.91, 0.97)	<0.001
Oppositional defiant problems	1.00 (0.97, 1.04)	0.872	0.97 (0.94, 1.00)	0.057	0.99 (0.95, 1.02)	0.498	1.01 (0.98, 1.05)	0.489	1.01 (0.98, 1.05)	0.348	1.02 (0.99, 1.05)	0.237

Notes: Adjusted for maternal age, gestational week, education, occupation, BMI in early pregnancy, anemia in mid-pregnancy, supplementation during pregnancy, delivery mode, children’s sex, age, birthweight, main caregiver, and kindergarten attendance.
